# Patient-derived organoids (PDOs): a novel preclinical platform to overcome challenges in cancer immunotherapy

**DOI:** 10.3389/fcell.2026.1757516

**Published:** 2026-02-18

**Authors:** Dongmei Ni, Junjie Xing, Gengming Niu, Jingjing Qiu, Guiying Wei, Gang Chen, Qinghe Zhou, Xiaolan Yin

**Affiliations:** 1 Cancer Center, Shanghai 411 Hospital, China RongTong Medical Healthcare Group Co., Ltd./411 Hospital, Shanghai University, Shanghai, China; 2 Department of Colorectal Surgery, Changhai Hospital, Naval Medical University, Shanghai, China; 3 Shanghai OneTar Biomedicine, Shanghai, China; 4 Jiaxing Key Laboratory of Basic Research and Clinical Translation on Orthopedic Biomaterials, Department of Orthopaedics, The Second Affiliated Hospital of Jiaxing University, Jiaxing, China; 5 Jiaxing Organoid Center, Jiaxing, China

**Keywords:** biomarker, cancer immunotherapy, immune checkpoint inhibitors (ICIs), patient-derived organoids (PDOs), tumor microenvironment (TME)

## Abstract

Cancer immunotherapy has revolutionized oncology but faces significant challenges including low response rates and lack of effective preclinical models. This review elucidates how patient-derived organoids (PDOs) are emerging as a transformative platform to address these hurdles. We detail sophisticated immuno-PDO (iPDO) models, categorized into reconstituted systems (co-culturing PDOs with exogenous immune cells) and native systems (preserving endogenous tumor microenvironment via Air-Liquid Interface or Patient-Derived Organotypic Tumor Spheroids). A problem-solution framework demonstrates how iPDOs: (1) deconvolute the immunosuppressive TME; (2) function as “living biomarkers” for predicting clinical responses; (3) unravel resistance mechanisms via multi-omics; and (4) empower high-throughput screening for personalized combination therapies. Integration with bioengineering, multi-omics, and AI heralds a new era in precision immuno-oncology, holding immense promise for deciphering resistance and improving clinical outcomes.

## 1 Introduction

### 1.1 The paradigm shift in cancer immunotherapy and its core challenges

**FIGURE 1:**
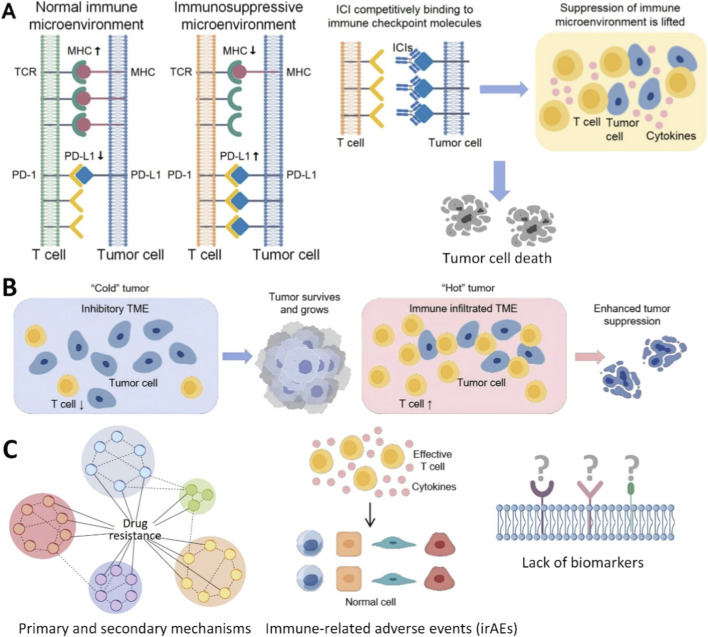
Cancer immunotherapy and the challenges it faces. **(A)** Overview of immunotherapy models. **(B)** The main factors restricting the efficacy of immunotherapy. **(C)** Major challenges for immunotherapy efficacy.

Cancer immunotherapy, particularly immune checkpoint inhibitors (ICIs), has revolutionized oncology by enabling durable clinical responses in a subset of patients through the reactivation of anti-tumor immunity (Munir et al., 2024; Chen et al., 2024; Sun S. et al., 2025; Bai and Cui, 2022). The mechanistic basis of ICIs, involving the blockade of inhibitory checkpoints such as PD-1 and CTLA-4 to rescue T-cell cytotoxicity, is schematically summarized in Figures 1A–C. However, the broad application of this revolutionary approach is severely constrained by several fundamental challenges: (1) low overall response rates, with primary resistance prevalent in “immunologically cold” tumors; (2) complex and heterogeneous mechanisms of primary and acquired resistance; (3) severe immune-related adverse events (irAEs); and (4) a critical lack of robust predictive biomarkers for precise patient stratification (Emens et al., 2024; Chong et al., 2024; Form et al., 2025) (Figure 1).

The root of these clinical hurdles lies in the profound heterogeneity and dynamic nature of the human tumor microenvironment (TME). A pivotal bottleneck in translating laboratory discoveries into clinical strategies, however, is the lack of preclinical models that faithfully recapitulate the heterogeneity and human-specific TIME interactions (Keenan et al., 2025; Zhou et al., 2024).

Traditional two-dimensional (2D) cell line models, while valuable for basic mechanistic studies, suffer from critical shortcomings that limit their relevance for immunotherapy research. Cultured as monolayers on plastic, they lose the original three-dimensional tissue architecture, cell-cell contacts, and extracellular matrix (ECM) interactions that are fundamental to *in vivo* tumor biology and drug response (Pampaloni et al., 2007). More critically, this simplified environment fails to recapitulate the complex spatial organization and biochemical gradients of the TME, which are essential for modeling immune cell infiltration, function, and exhaustion (Wi et al., 2014). Long-term *in vitro* passaging also leads to genetic and phenotypic drift, selecting for clones adapted to plastic rather than preserving the heterogeneous landscape of a patient’s tumor (Wi et al., 2014; Gillet et al., 2013). Consequently, 2D models are inherently inadequate for studying the dynamic, multicellular interactions that underly immunotherapy efficacy, resistance, and toxicity.

While patient-derived xenograft (PDX) models retain key aspects of tumor heterogeneity and architecture (Hidalgo et al., 2014), their utility in immuno-oncology is fundamentally limited by the interspecies barrier (Gu et al., 2025). Conventional PDX models are established in immunodeficient mice, which not only lack a functional immune system but also, upon engraftment, undergo a gradual replacement of human stromal components with murine counterparts. This species mismatch disrupts the critical, human-specific cytokine and cell-cell signaling networks that govern tumor-immune interactions (Blomme et al., 2018; Yoshida, 2020; Mosmann et al., 1987). Consequently, conventional PDXs are unsuitable for studying the efficacy and resistance mechanisms of human immunotherapies or for modeling immune-related toxicity. Although efforts to create humanized mouse models by engrafting human immune cells (e.g., CD34^+^ hematopoietic stem cells) aim to bridge this gap, they introduce complexities such as graft-versus-host disease and may not fully recapitulate the patient’s native immune ecosystem (Chiorazzi et al., 2023). These inherent limitations underscore the urgent need for a purely *human*, patient-specific *ex vivo* platform to faithfully model the TME.

### 1.2 Patient-derived organoids: a next-generation bridge to precision immuno-oncology

Patient-derived organoids (PDOs) are self-organizing, three-dimensional, organ-like structures cultured *ex vivo* from patient tumor tissue (Praharaj et al., 2018; Sato et al., 2009). Compared to conventional models, PDOs exhibit high fidelity to the original tumor in terms of genetics, histopathology, and drug response profiles (Verduin et al., 2023; Han et al., 2024; Vlachogiannis et al., 2018). Crucially, by co-culturing PDOs with autologous immune cells (generating “immuno-PDOs” or iPDOs) or employing specialized culture methods (e.g., Air-Liquid Interface) to preserve endogenous tumor-infiltrating immune cells, researchers can now reconstruct a functional, patient-specific TME *in vitro* (Polak et al., 2024; Dijkstra et al., 2018; Neal et al., 2018).

This review aims to systematically elucidate how iPDOs serve as a transformative platform to directly address the core clinical challenges outlined above. We propose a “problem-solution” framework detailing how iPDOs are being leveraged to: (1) deconvolute the immunosuppressive TME; (2) function as dynamic “living biomarkers” for response prediction; (3) unravel mechanisms of immunotherapy resistance; and (4) enable high-throughput screening for personalized combination therapies. Finally, we discuss current limitations and future perspectives on integrating iPDOs with bioengineering and multi-omics to usher in a new era of precision immuno-oncology.

## 2 PDOs: a next-generation preclinical model

### 2.1 Fundamentals of 3D ex vivo culture

The research on organoids dates back to 2009. Hans Clevers et al. first utilized mice LGR5^+^ intestinal stem cells to self - organize *in vitro* into intestinal organoids featuring the crypt - villus structure (Sato et al., 2009). Subsequently, this paradigm has been successfully extended to a wide range of tissues, including cancers (Praharaj et al., 2018), providing a stable and expandable *ex vivo* platform that bridges the gap between simplistic cell lines and complex *in vivo* models.

### 2.2 Advantages of PDOs as a platform for immuno-oncology

Compared to conventional preclinical models, PDOs offer a unique combination of fidelity, scalability, and experimental tractability that makes them particularly suited for immuno-oncology research (Table 1).

**TABLE 1:** Comparison of major iPDO modeling strategies.

Feature	Reconstituted Co-culture models	Native ALI-PDOs	Native PDOTS
Starting material	Pre-established PDOs + added immune cells	Minced tumor fragments	Partially digested tumor fragments
Immune compartment	Defined, exogenous input	Endogenous, diverse populations	Endogenous, diverse populations
Spatial architecture	Disrupted; re-established in co-culture	Preserved native architecture	Partially preserved
Culture duration	Long-term	Long-term (>70 days)	Short-term (1–2 weeks)
Throughput	Moderate to high	Low	Moderate (enabled by microfluidics)
Key advantages	• High flexibility and modularity • Suitable for genetic engineering and high-throughput screening • Enables study of specific immune subsets	• Most faithful retention of original TIME • Long-term culture of native immune cells • Preserves TCR repertoire	• Retains autologous immune and stromal cells • Suitable for dynamic drug testing • Faster establishment than ALI
Primary limitations	• Lacks native stromal and immune context • May introduce non-physiological interactions	• Low throughput • Technically challenging • Genetic manipulation is difficult	• Shorter culture duration • Architecture partially disrupted by digestion

High Patient-Specific Fidelity: PDOs maintain the genetic, transcriptomic, and histological heterogeneity of the parental tumor. Whole-exome sequencing studies have shown mutation retention rates exceeding 90% in glioblastoma and other cancers, faithfully preserving driver alterations (Verduin et al., 2023; Han et al., 2024). Crucially, this genetic fidelity translates to functional fidelity in drug response. Landmark studies in gastrointestinal cancers have demonstrated a strong correlation between PDO drug sensitivity *in vitro* and patient clinical outcomes, establishing PDOs as a predictive pharmacotyping tool (Vlachogiannis et al., 2018).

Recapitulation of the Tumor Microenvironment Architecture: Unlike 2D monolayers, PDOs grow in three dimensions, preserving cell-cell interactions, polarity, and gradients of signaling molecules. This architecture is fundamental for studying processes like immune cell infiltration and function, which are lost in traditional 2D culture (Pampaloni et al., 2007; Boj et al., 2015).

A Purely Human, Scalable System: PDOs circumvent the interspecies barrier that limits PDX models in immunotherapy research. As a purely human *ex vivo* system, they avoid the gradual replacement of human stroma with murine components and the resultant disruption of human-specific cytokine networks (Gu et al., 2025; Yoshida, 2020). Furthermore, PDOs exhibit faster establishment times and higher success rates compared to PDXs, enabling the creation of large, clinically annotated biobanks (Li et al., 2023). Their scalability supports medium-to high-throughput drug screening, which is impractical in animal models (Ding et al., 2022).

Facilitates Immune Integration: The most significant advancement for immunotherapy is the ability to generate iPDOs. This is achieved either by reconstituting established PDOs with autologous immune cells (e.g., T cells, CAR-T cells) or by preserving the native endogenous immune niche using specialized methods like the Air-Liquid Interface (ALI) (Polak et al., 2024; Dijkstra et al., 2018; Neal et al., 2018). This capability directly addresses the core deficiency of previous models by enabling the study of dynamic, human-specific tumor-immune interactions *in vitro*.

Therefore, PDOs represent a transformative preclinical platform that balances physiological relevance with experimental control. By integrating a functional human immune component, iPDOs are uniquely positioned to address the persistent challenges in cancer immunotherapy, as detailed in the following problem-solution framework (Section 3).

## 3 Addressing immunotherapy challenges with PDOs: a problem-solution framework

### 3.1 Challenge 1: deconvoluting the complex tumor microenvironment (TME)

Problem: The immunosuppressive TME (e.g., T-cell exhaustion, Tregs, M2 macrophages) is a key driver of immunotherapy resistance (Sharma et al., 2017). However, this complexity is poorly modeled in traditional systems (Pampaloni et al., 2007; Hidalgo et al., 2014), such as 2D cell lines and PDXs in immunodeficient mice, which lack a functional human immune context.

PDO Solution: Engineering Next-Generation iPDOs.

**FIGURE 2:**
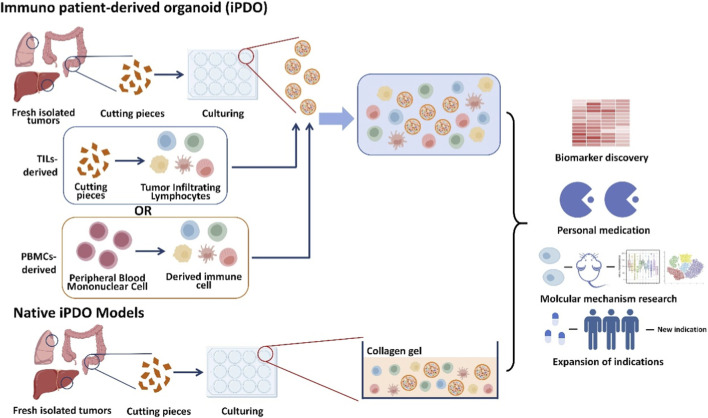
Workflow for establishing and applying immuno-PDO (iPDO) models in cancer immunotherapy research.

To bridge this gap, the field has developed sophisticated PDO models that incorporate immune components, collectively termed iPDOs. These models can be broadly categorized into reconstituted and native systems (Polak et al., 2024), each with distinct advantages and applications (Table 1) (Figure 2).

#### 3.1.1 Reconstituted iPDO models: introducing immunity into established PDOs

This approach involves co-culturing pre-established, epithelial-only PDOs with various sources of immune cells (Polak et al., 2024). Its primary advantage is flexibility and modularity, allowing for the study of specific immune-tumor interactions.

Sources of Immune Cells: PDOs can be co-cultured with: a)Autologous immune cells (Dijkstra et al., 2018), such as peripheral blood mononuclear cells (PBMCs) or isolated T cells from the same patient; b)Tumor-infiltrating lymphocytes (TILs) (Neal et al., 2018), isolated and expanded from the patient’s tumor tissue; and c)Engineered immune cells (Logun et al., 2025; Lin et al., 2025), such as chimeric antigen receptor (CAR)-T cells and CAR-natural killer (NK) cells.

Applications and Evidence: This platform enables real-time study of human-specific immune-tumor interactions. For instance, co-culture of colorectal cancer (CRC) or non-small cell lung cancer (NSCLC) PDOs with autologous PBMCs can generate and expand tumor-reactive T cells (Dijkstra et al., 2018; Cattaneo et al., 2020). Similarly, the introduction of CAR-T cells into PDO co-culture systems allows for the direct visualization of tumor cell killing and the investigation of CAR-T cell dysfunction (Dekkers et al., 2023).

#### 3.1.2 Native iPDO models: preserving the endogenous immune niche

Unlike reconstituted models, native iPDO approaches initiate culture from tumor tissue fragments with minimal disruption, thereby preserving the original tumor architecture, stromal components, and endogenous immune cell populations (Polak et al., 2024). These models offer a more holistic view of the TME.

ALI Method: In this model, physically minced tumor fragments are embedded in a collagen gel at an ALI, which promotes sufficient oxygenation and nutrient diffusion (Neal et al., 2018; Ootani et al., 2009). ALI-PDOs can be cultured long-term (>70 days) and maintain a diverse array of endogenous immune cells, including T cells, B cells, NK cells, and tumor-associated macrophages (TAMs). Critically, they preserve the original T-cell receptor (TCR) repertoire of the patient’s TILs, enabling the evaluation of patient-specific responses to ICIs *in vitro* (Neal et al., 2018; Esser et al., 2020).

Patient-Derived Organotypic Tumor Spheroids (PDOTS): This microfluidic-based method cultures small tumor fragments (40–100 μm in diameter) within a collagen matrix flanked by media channels (Jenkins et al., 2018). PDOTS retain autologous lymphocytes and myeloid cells for 1–2 weeks and have been successfully used to model dynamic responses to ICIs and to identify novel therapeutic combinations that can overcome resistance, such as CDK4/6 or TBK1 inhibitors (Jenkins et al., 2018; Deng et al., 2018; Sun et al., 2023).

#### 3.1.3 Insights into specific immune cell behaviors from iPDOs

Leveraging these iPDO models has yielded mechanistic insights into TIME dynamics. For instance, effector T cells induce organoid apoptosis via direct contact and release of cytokines (IFN-γ, TNF-α) (Wang et al., 2025), while B cells can enhance anti-tumor immunity in organoid models by forming tertiary lymphoid structure (TLS)-like structures and activating CD4^+^ T cells via MHC-II presentation (Wang et al., 2025; Helmink et al., 2020). Neutrophils exhibit a dual role, being co-opted to promote tumor cell migration and metastasis in organoid co-culture models by releasing neutrophil extracellular traps (NETs), proteases like MMP-9, and NE (Wang et al., 2025). Furthermore, NK cells demonstrate potent cytotoxicity against tumor organoids via receptors like NKG2D, leading to the release of Granzyme B and IFN-γ. Strategies combining NK cells with novel engagers (e.g., TriKE) have shown enhanced tumor-killing efficacy in microfluidic organoid systems (Wang et al., 2025).

### 3.2 Challenge 2: identifying predictive biomarkers of response

Problem: Static biomarkers like PD-L1 expression and tumor mutational burden (TMB) have inconsistent predictive power and fail to capture the dynamic functional state of the tumor-immune interaction (Hav et al., 2019).

PDO Solution: The PDO-based Functional Diagnostic as a “Living Biomarker”.

PDOs offer a paradigm shift from static, single-parameter biomarkers to a dynamic, integrated functional readout. By employing the iPDO platforms described in Section 3.1, it is possible to create a “living biomarker” – a personalized *ex vivo* model that directly tests the efficacy of immunotherapies on the patient’s own tumor within its immune context.

#### 3.2.1 Recapitulating patient-specific responses to immunotherapy

The predictive validity of iPDOs is demonstrated by their correlation with clinical outcomes.

In Reconstituted Models: The magnitude of tumor organoid killing by co-cultured autologous T cells or CAR-T cells has been shown to correlate with clinical response to corresponding therapies (Shang et al., 2024; Schnalzger et al., 2019).

In Native Models: patient-specific T-cell activation and tumor killing upon anti-PD-1/PD-L1 treatment *in vitro* mirrored the patients’ subsequent clinical responses to ICIs (Neal et al., 2018).

#### 3.2.2 Enabling high-throughput and multiplexed readouts

A key advantage is the ability to generate rich, multidimensional data from a single assay.

Quantifiable Endpoints: When iPDOs are exposed to ICIs or other immunotherapies, a suite of analytical readouts can be employed (For et al., 2021; Olawade et al., 2025; Cao et al., 2025; Liu et al., 2025; Dong et al., 2023; Ren et al., 2025): a)Tumor Cell Killing, measured by caspase activation, live/dead staining, ATP activity, or organoid size quantification; b)Immune Cell Activation and Proliferation, analyzed via flow cytometry for surface activation markers (e.g., BTN3A1 and BTN2A1) and intracellular cytokines/enzymes (e.g., IFN-γ, TNF-α, Granzyme B); c)Immune Cell Phenotype and Clonality, assessed using single-cell RNA sequencing (scRNA-seq) to track clonal expansion and exhaustion states.

#### 3.2.3 Guiding rational combination therapies

iPDOs enable empirical testing of combination strategies directly on patient tissue to overcome single-agent resistance. Systematic Screening: The miniaturized format of PDOs, particularly in reconstituted co-culture or micro-organosphere (MOS) systems, allows for high-throughput screening of dozens of drug combinations (e.g., ICI + targeted therapy, ICI + chemotherapy, ICI + novel immunomodulators) (Ding et al., 2022).

Mechanism-Driven Discovery: This approach has successfully identified synergistic combinations. For instance, screening using PDOTS identified that inhibitors of CDK4/6 can enhance T-cell activation and synergize with ICIs (Deng et al., 2018). Similarly, TBK1 inhibition was discovered to overcome ICI resistance in PDOTS models of melanoma and CRC (Sun et al., 2023).

### 3.3 Challenge 3: unraveling mechanisms of primary and acquired resistance

Problem: Resistance to immunotherapy is a major clinical setback, driven by highly heterogeneous and dynamic tumor-intrinsic and -extrinsic mechanisms. Dissecting these complex, often patient-specific, pathways in traditional models is challenging, hindering the development of effective countermeasures (Sharma et al., 2017).

PDO Solution: A High-Definition Platform for Mechanistic Discovery

iPDOs provide a genetically and phenotypically faithful *ex vivo* system to functionally dissect resistance mechanisms. By applying sophisticated perturbations and multi-omics analyses directly to patient-derived tissue, iPDOs can move beyond correlation to establish causality (Verdys et al., 2025; Yang et al., 2025; Lee et al., 2025).

#### 3.3.1 Functional genomics and CRISPR screening

The genetic tractability of PDOs allows for systematic, genome-scale interrogation of gene function in a native human tumor context.

Identifying Key Evasion Genes: CRISPR-Cas9-based knockout screens in PDOs co-cultured with immune cells can pinpoint genes essential for immune evasion. For instance, *in vivo* CRISPR screens have identified genes that, when knocked out, sensitize tumors to T-cell attack, a methodology directly adaptable to iPDOs (Dubrot et al., 2022). Modeling Specific Resistance Pathways: Beyond screening, CRISPR-Cas9 can be used to introduce specific mutations found in non-responders into sensitive PDOs, or *vice versa*, to validate their functional role in driving resistance (Dubrot et al., 2022).

#### 3.3.2 Multi-omics deconvolution of the resistant TME

Comparative analysis of sensitive versus resistant iPDOs reveals the molecular underpinnings of treatment failure.

Single-Cell and Spatial Profiling: Applying single-cell RNA sequencing (scRNA-seq) to iPDOs can dissect how therapy reshapes the entire cellular ecosystem, uncovering shifts in T-cell exhaustion states or the emergence of immunosuppressive populations (Pelk et al., 2021). Integrating this with spatial transcriptomics or multiplexed imaging (e.g., CODEX, MIBI) can further reveal the critical cellular neighborhoods that foster resistance (Phillips et al., 2021; Schürch et al., 2020).

TCR Clonotype Tracking: In native iPDO models like ALI-PDOs, sequencing can track the fate of specific T-cell clones upon treatment to determine if resistance is due to clonal failure or exclusion (Qin et al., 2025; Sun H. et al., 2025).

#### 3.3.3 Modeling stromal contributions

iPDO models enable direct study of non-cell-autonomous mechanisms of resistance. Studying Fibroblast-Immune Crosstalk: Co-culture of PDOs with cancer-associated fibroblasts (CAFs) has demonstrated that CAFs-derived factors can directly suppress T-cell functio (Seino et al., 2018; Strating et al., 2023).

### 3.4 Challenge 4: developing and optimizing rational combination therapies

Problem: With a vast and growing arsenal of anticancer agents, identifying the most effective and tolerable ICI-based combination for an individual patient is a monumental clinical challenge (Sharma et al., 2023).

PDO Solution: High-Throughput Personalized Combination Screening.

The miniaturization, scalability, and fidelity of PDOs make them an ideal platform for performing empirical, high-throughput drug screening directly on patient tissue (Polak et al., 2024).

#### 3.4.1 Systematic in vitro clinical trials

PDO biobanks, representing a spectrum of cancer subtypes and molecular backgrounds, can be used to systematically test novel immunotherapy combinations *in vitro*.

Identifying Synergistic Combos: By screening libraries across a large panel of PDOs, researchers can identify combos effective in specific molecular subtypes (Ding et al., 2022).

Prioritizing Clinical Candidates: This “Phase 0” screening approach de-risks drug development and has been successfully used to identify and validate combinations like CDK4/6i + ICIs and TBK1i + ICIs (Jenkins et al., 2018; Deng et al., 2018; Sun et al., 2023).

#### 3.4.2 Guiding personalized combination regimens

For patients with advanced, treatment-resistant disease, iPDOs can be used to create a personalized treatment recommendation in real time.

The “Functional Diagnostic” for Combinations: iPDOs can test a bespoke panel of 2- or 3-drug combinations tailored to the patient’s tumor. This is particularly valuable for navigating complex strategies like combining ICIs with anti-angiogenics or next-generation bispecific antibodies (Wu et al., 2025; Kang et al., 2025). Beyond Pharmaceuticals: The flexibility of iPDOs extends beyond pharmaceuticals to assess synergy with adoptive cell therapies (e.g., CAR-T, TILs) (e.g., CAR-T, TILs) (Schnalzger et al., 2019; Ning et al., 2024) or modulation of the microbiome (Zitvogel et al., 2018).

#### 3.4.3 Uncovering novel mechanisms of synergy

The iPDO platform is not just a screening tool but also a discovery engine that can reveal the biological mechanisms underlying effective combinations.

From Observation to Mechanism: When a synergistic drug pair is identified in a screen, the same iPDO model can be immediately subjected to the multi-omics analyses to understand *why* it works (Sun et al., 2023).

## 4 Current limitations and future perspectives

**FIGURE 3:**
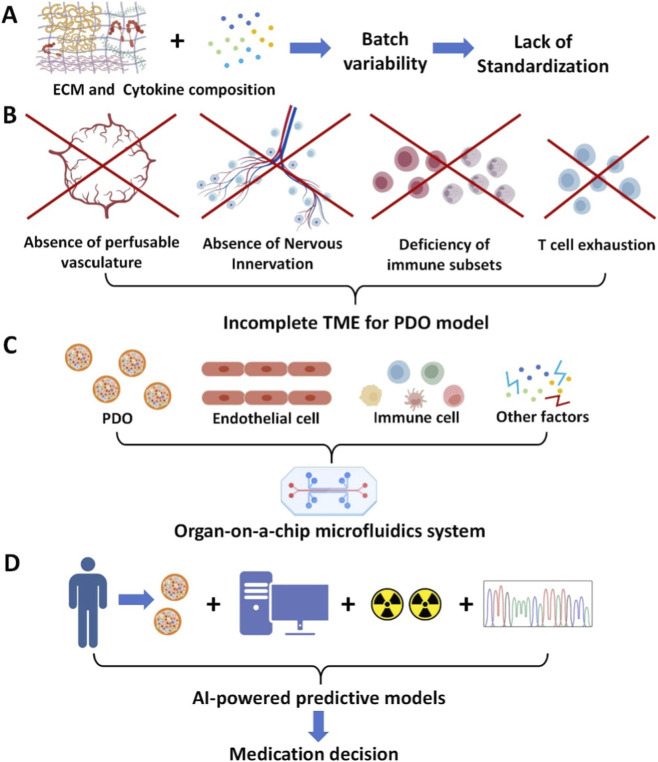
Major limitations and next-generation application directions for iPDO models. **(A)** Major limitations faced in the standardization of iPDO construction. **(B)** The existing iPDO models are supplied with inadequate microenvironment components. **(C)** The cross-application of the traditional PDO models and microfluidic technology. **(D)** The iPDO model has been further integrated into a more complete artificial intelligence model. Abbreviations: PDO, patient-derived organoid; iPDO, immune-PDO.

Despite the transformative potential of iPDOs, their translation into routine clinical decision-making and drug discovery is contingent upon overcoming significant technical, biological, and translational hurdles. A clear-eyed view of these limitations charts the course for future innovation (Figure 3).

### 4.1 Persistent technical and biological hurdles

#### 4.1.1 Standardization, scalability, and ECM variability

Technical Variability in iPDOs Generation: Variability stems from differences in stem cell lines, donor heterogeneity, culture protocols, and operator expertise, leading to inconsistent differentiation efficiencies and functional outputs across laboratories (Rezvani et al., 2025).

Emerging Solutions: Addressing technical variability demands a systematic, multi-layered strategy spanning protocol standardization, material optimization, and advanced monitoring. Essential foundational steps include establishing transparent reporting standards for critical parameters—such as extracellular matrix lot numbers—and validating protocols across laboratories to ensure consistency. Concurrently, the field is transitioning toward chemically defined systems, including xeno-free culture media and tunable hydrogels, which offer improved batch-to-batch reproducibility while maintaining physiological relevance. Real-time quality control via flow cytometry and single-cell RNA sequencing enables dynamic monitoring of differentiation and cellular states. Further refinement is achieved through automated culture systems and engineered microenvironments using techniques such as micropatterning and sequential crosslinking, which reduce operator-dependent variation and enhance spatial precision, ultimately supporting reproducible, scalable disease modeling and therapeutic screening (Rezvani et al., 2025; Rauner et al., 2025).

ECM Batch Variability: Widely used natural matrices, such as Matrigel (a basement membrane extract from mouse sarcoma), are complex, ill-defined mixtures containing over 2,000 proteins and 14,000 unique peptides (Hughes et al., 2010). This composition leads to significant batch-to-batch variability, the presence of xenogenic contaminants, and limited tunability of biochemical and mechanical properties. These factors unpredictably influence organoid phenotype, hinder reproducibility, and pose barriers to clinical application (Li et al., 2025; Aisenbrey and Murphy, 2020).

Emerging Solutions: To address these issues, the field is shifting towards engineered/synthetic matrices. Chemically defined systems, such as polyethylene glycol (PEG)-based hydrogels, recombinant protein scaffolds (e.g., elastin-like proteins), and hybrid polymers, offer precise control over ligand presentation, stiffness, porosity, and degradation rates (Gjorevski et al., 2016; Cruz-Acuña et al., 2017; Broguiere et al., 2018). These matrices provide high batch-to-batch reproducibility, enable the mimicry of patient-specific ECM niches, and are better suited for scalable, high-throughput drug screening applications (Li et al., 2025; Hunt et al., 2021).

#### 4.1.2 An incomplete TME

While iPDOs represent a leap forward, they remain a simplified model. Key components of the *in vivo* reality are absent or poorly represented:

Lack of Functional Vasculature: The absence of functional vasculature represents a critical limitation in current iPDOs, particularly when modeling tumor‐immune interactions and immunotherapy responses. As highlighted in vascular organoid research, physiological relevance in 3D models depends heavily on the integration of vascular networks to support nutrient diffusion, oxygen supply, waste removal, and immune cell trafficking. In conventional iPDO systems, the lack of perfusable vasculature leads to hypoxic cores, necrotic regions, and limited immune cell infiltration—factors that poorly replicate the dynamic TME where endothelial cells regulate immune cell recruitment and activation (Naderi-Meshkin et al., 2023; Zhou et al., 2025).

Emerging Solutions: Strategies to address this limitation are advancing along two primary paradigms: biological self-organization and engineered pre-vascularization. The biological approach typically involves co-culturing organoids with endothelial cells and/or supplementing with pro-angiogenic factors to stimulate intrinsic vascular network formation. Alternatively, engineering strategies utilize platforms like microfluidic organ-on-a-chip systems and advanced biomaterial scaffolds to create perfusable, pre-formed vascular architectures that can be integrated with organoids. These solutions aim to recapitulate critical vasculature-dependent processes—such as immune cell extravasation and endothelial-immune crosstalk—thereby enhancing the physiological relevance and predictive value of iPDOs for immunotherapy research (Zhou et al., 2025; Lai et al., 2021; Xiao et al., 2025; Rajasekar et al., 2020).

Lack of humoral immunity components: Existing models for immunotoxicity and immunogenicity testing face significant limitations in recapitulating the complex microenvironment required for humoral immune responses. Simple *in vitro* cell cultures, such as suspended PBMCs or monolayer co-cultures, are designed only for short-term, static conditions and lack the tissue functionality and organ physiology necessary to support processes like B cell activation, germinal center formation, and antigen-specific antibody production. This gap hinders the reliable evaluation of vaccine efficacy, therapeutic antibody functionality, and drug-induced humoral immunotoxicity in a human-relevant context (Giese et al., 2010).

Emerging Solutions: The development of a human artificial lymph node (HuALN) model, implemented in a miniaturized, perfused bioreactor system, provides a novel solution. This 3D micro-organoid culture system combines autologous PBMCs—including B cells, T cells, and antigen-presenting dendritic cells—within a macro-porous agarose matrix under controlled perfusion. This setup enables long-term culture (14–30 days) and supports the self-organization of lymphoid structures. The model successfully demonstrates key features of adaptive immunity: antigen-specific B cell activation, plasma cell differentiation, and the secretion of immunoglobulins (IgM, with indications of class switching). It allows for the monitoring of both cellular (via cytokine profiles) and humoral (via antibody secretion) immune responses to vaccines (e.g., Hepatitis A) or viral antigens (e.g., CMV), offering a physiologically relevant *in vitro* platform for immunological substance testing (Giese et al., 2010).

Myeloid vs. Lymphoid Lineage Imbalance in Immune Organoids: A common limitation of current immune-organoid systems is their skewed lineage output, with a predominant bias toward myeloid cell differentiation—such as macrophages and granulocytes—while lymphoid lineages (B and T cells) are often underrepresented or require extensive exogenous induction (Rezvani et al., 2025). This imbalance restricts the modeling of adaptive immune responses and lymphocyte-mediated immunotherapies within organoid platforms.

Emerging Solutions: To enhance lymphoid development, strategies include supplementing culture systems with lymphoid-specific cytokines (e.g., IL-7, FLT3L) and incorporating stromal co-cultures that provide Notch signaling and other niche factors essential for lymphocyte maturation. Small molecules such as UM171 have also been employed to expand multipotent progenitors with enhanced lymphoid potential. Further engineering of the organoid microenvironment—through vascularization or integration of lymphoid-like niche structures—holds promise for establishing more balanced and functional immune lineage representation (Rezvani et al., 2025).

Immune Cell Attrition and Stromal Loss: A fundamental limitation of widespread organoid culture methods, particularly the submerged Matrigel culture system, is the systematic loss of non-epithelial components during establishment. During the enzymatic and physical dissociation of tumor tissue, followed by selective culture in niche factor-enriched media, the native tumor microenvironment is stripped away. This results in organoid cultures that are highly enriched for epithelial cancer cells but devoid of the resident immune infiltrate (such as T cells, B cells, macrophages, and dendritic cells) and critical stromal populations like CAFs. Consequently, these “immune-empty” and “stroma-deficient” organoids fail to model the complex paracrine and juxtacrine signaling networks between cancer cells, immune cells, and the stroma that are pivotal for tumor progression, drug resistance, and response to immunotherapies (as reviewed in (Xia et al., 2021)). This attrition fundamentally limits their utility in studying immune checkpoint blockade, adoptive cell therapies, and stromal-targeted interventions.

Emerging Solutions: The reconstruction of physiologically relevant tumor-stroma interactions is being advanced through layered experimental strategies. A foundational approach involves co-culturing organoids with primary stromal components such as CAFs, which has been shown to potentiate tumor cell proliferation and invasive capacity. To better preserve native tissue architecture, ALI culture systems provide a more physiological microenvironment for stromal maintenance and function. For higher-order integration, microfluidic tumor-on-a-chip platforms enable the incorporation of stromal elements within dynamically perfused systems, where controlled fluid flow and mechanical forces help establish complex, organ-level interactions that more accurately mimic *in vivo* conditions (Rauner et al., 2025).

Selective Pressure and Clonal Representation: The process of deriving and expanding PDOs *in vitro* can apply selective pressure, potentially favoring the growth of specific subclones over others. This may lead to PDOs that does not fully capture the intra-tumoral heterogeneity of the original tumor, especially rare, treatment-resistant clones that ultimately drive clinical relapse (Beshiri et al., 2023).

*Emerging Solutions*: The tissue fragment-based culture methodology represents a key solution by avoiding enzymatic digestion and preserving the original multicellular architecture. This approach significantly reduces the selective pressure that favors the outgrowth of dominant clones in conventional models, thereby maintaining a more authentic representation of the tumor’s cellular and clonal diversity. By embedding mechanically minced fragments directly into a supportive ECM, this strategy better recapitulates the complex cell-cell and cell-matrix interactions critical for preserving native heterogeneity (Zheng et al., 2025).

Long-Term Maintenance of Immune Compartment: Sustaining functional immune cells, particularly T cells, in co-culture beyond 1–2 weeks remains challenging. While supplementation with cytokines like IL-2 can help, it may also drive non-physiological T-cell activation or exhaustion, altering the native immune state (Neal et al., 2018).

Emerging Solutions: Preserving functional immune ecosystems in tumor models relies on optimized culture systems that balance stability and physiological relevance. Static, matrix-embedded platforms maintain native immune-tumor interactions by minimizing shear stress and supporting diverse immune populations, while high-throughput micro-organospheres and vascularized organ-on-chip systems enable scalable screening and dynamic modeling of immune trafficking and therapeutic responses. Together, these approaches provide durable, patient-specific platforms for immunotherapy evaluation and immune-oncology research (Rauner et al., 2025; Zheng et al., 2025).

Lack of mechanical and metabolic cues: Current organoid models, largely dependent on reconstituted basement membrane (rBM), fail to recapitulate critical *in vivo* mechanical forces (e.g., compression, tension, shear stress) and physiological metabolic gradients. The globular structure of rBM limits cell-matrix interactions essential for migration and invasion, while its composition lacks key fibrillar ECM proteins like collagen I/III that govern tissue mechanics. This simplification impedes accurate modeling of mechanotransduction, metabolic stress responses, and associated therapeutic resistance (as reviewed in (Rauner et al., 2025)).

Emerging Solutions: Emerging strategies focus on advanced biomaterials, engineered culture systems, and integrated analytics to restore mechanical and metabolic fidelity. These include employing tunable natural/synthetic hydrogels to control stiffness and architecture, utilizing microfluidic tumor-on-a-chip platforms to introduce perfusion and shear stress, and applying ALI cultures with collagen matrices to improve gas exchange and structural maturation. Coupled with high-resolution live imaging and AI-driven analysis, these approaches enable dynamic quantification of cellular responses to biomechanical and metabolic cues within 3D microenvironments, enhancing physiological relevance and predictive capacity for cancer research (Neal et al., 2018; Rauner et al., 2025; Urciuolo et al., 2023; Nishiguchi et al., 2018).

### 4.2 Translational applications and next frontiers

Addressing the above limitations is an active area of research. Emerging strategies aim to enhance the physiological relevance and clinical utility of iPDOs through technological integration and novel applications.

#### 4.2.1 Enhanced bioengineering for next-generation iPDOs

The convergence of iPDOs with advanced engineering is creating more complex and physiologically accurate models. Organ-on-a-chip microfluidics and 3D bioprinting enable the incorporation of perfusable vasculature, multi-tissue interfaces (e.g., for metastasis or toxicity studies), and dynamic control over biophysical cues (Brassard et al., 2021; Ingber, 2022). The development of defined synthetic matrices aims to replace animal-derived substrates, reducing batch variability and allowing precise tuning of the mechanical and biochemical niche (Gjorevski et al., 2016). These advancements directly address the limitations of TME incompleteness and standardization.

#### 4.2.2 Guiding clinical trials and the “Digital Twin” concept

iPDO biobanks can be used to stratify patients in innovative clinical trial designs, such as “umbrella” or “basket” trials. The vision of a “Digital Twin”—where a patient’s iPDO response data is integrated with their clinical, genomic, and radiomic profiles to build AI-powered predictive models—could revolutionize treatment decision-making and outcome prediction.

#### 4.2.3 Enabling personalized immunotherapy development

iPDOs serve as a dual-purpose platform for developing and testing bespoke immunotherapies. They can be used to identify patient-specific neoantigens and subsequently to functionally validate the efficacy of corresponding vaccines or adoptive cell therapies (e.g., neoantigen-specific T cells) *ex vivo* before patient administration (Hu et al., 2024; Huang et al., 2022). This closes the loop between antigen discovery and therapeutic assessment in a patient-specific context.

#### 4.2.4 Modeling systemic immunity and the microbiome

Future models seek to recapitulate broader physiological systems to study immunity holistically. Coupling iPDOs with lymphoid organoids (e.g., lymph node or tonsil organoids) could model the critical antigen-presentation and T-cell priming phase (Wagar et al., 2021). Furthermore, incorporating patient-derived gut microbiota or their metabolites into iPDO cultures offers a pathway to mechanistically dissect and harness the gut-tumor-immune axis, which significantly influences immunotherapy efficacy and toxicity (Gopalakrishnan et al., 2018; Baruch et al., 2021).

### 4.3 Remaining unknowns and controversies

While iPDOs represent a significant advance in modeling human tumor-immune interactions, several fundamental questions remain unresolved, and their implications for clinical translation warrant careful scrutiny.

How Faithfully Do iPDOs Preserve the Patient’s Native Immune Context?

Although methods like ALI and PDOTS aim to retain endogenous immune cells, the extent to which the *in vitro* immune repertoire reflects the original tumor immune landscape is not fully established. Studies often report a reduction in T-cell clonality or shifts in immune subset proportions over time, raising concerns about whether iPDOs truly capture the functional diversity and spatial organization of the original TIME.

What Is the Functional Half-Life of Immune Cells in iPDO Cultures?

Most native iPDO models support immune cell viability for 1–3 weeks, but functional persistence—especially of cytotoxic T cells and antigen-presenting cells—remains limited. Cytokine supplementation (e.g., IL-2) can extend survival but may induce non-physiological activation or exhaustion. The lack of long-term immune maintenance hampers studies of chronic immune modulation and acquired resistance.

How Generalizable Are iPDO Responses Across Tumor Types and Patients?

PDO establishment success rates vary widely across cancer types, potentially biasing the applicability of iPDO-based findings. For instance, while colorectal and gastric cancer PDOs can often be established with high efficiency (>80% with our own experience), more challenging tumor types such as pancreatic cancer and sarcoma typically exhibit lower success rates (<50% with our own experience) due to factors like stromal complexity, low cellularity, or specific niche factor requirements. This variability highlights a selection bias that may limit the generalizability of conclusions drawn from iPDO studies, particularly for tumors that are inherently difficult to culture *ex vivo*. Moreover, the correlation between iPDO drug response and clinical outcome, while promising in selected studies, has not been validated in large, prospective multi-center trials. Variability in culture protocols, immune cell sources, and readout assays further complicates cross-study comparisons.

Do iPDOs Adequately Model Systemic Immune Effects?

Current iPDO systems are inherently local, focusing on the tumor-immune interface. They do not capture systemic immune dynamics—such as priming in lymph nodes, circulating immune cell trafficking, or distal immune-related adverse events—which are critical for understanding immunotherapy efficacy and toxicity *in vivo*.

Can iPDOs Predict Response to Combination Therapies in Real-Time Clinical Settings?

While high-throughput screening in iPDOs holds promise for personalized therapy selection, the turnaround time (often 2–4 weeks for PDO establishment and drug testing) may not align with the urgent clinical needs of patients with advanced disease. Additionally, the cost and scalability of iPDO generation remain barriers to routine clinical implementation.

Addressing these open questions requires concerted efforts in protocol standardization, longitudinal multi-omics tracking, and robust clinical validation studies. Only through rigorous, transparent, and collaborative science can iPDOs transition from a promising preclinical tool to a reliable component of precision immuno-oncology.

## 5 Conclusion

Cancer immunotherapy has irrevocably altered the oncology landscape, offering the potential for durable remission. However, its broad success is hampered by the dual challenges of low response rates and a lack of biomarkers to guide its application.

In this review, we have argued that iPDOs represent a transformative preclinical platform poised to address these hurdles. iPDOs effectively bridge the critical gap between simplistic 2D cell lines and complex, costly, and often non-human *in vivo* models. They retain the genetic and phenotypic fidelity of the parent tumor and, for the first time, allow for the direct and dynamic study of human tumor-immune interactions in an experimentally tractable system.

The iPDO toolkit—encompassing both reconstituted and native approaches—enables a multifaceted assault on the central problems in immuno-oncology:

- They deconvolute the complex tumor immune microenvironment (Challenge 1).
- They function as a “living biomarker” to stratify patients likely to respond to immunotherapy (Challenge 2).
- They provide a high-resolution platform for dissecting the mechanistic basis of primary and acquired resistance (Challenge 3).
- They empower high-throughput, personalized screening to identify the most effective rational combination therapies (Challenge 4).

While technical challenges surrounding standardization and TME completeness remain, the trajectory of innovation is clear. The ongoing integration of iPDOs with advanced bioengineering, multi-omics technologies, and artificial intelligence heralds a new era of precision immuno-oncology. By enabling functional drug testing and mechanistic studies directly on patient tissue, iPDOs hold the immense promise of deciphering resistance, personalizing combination immunotherapy, and ultimately, improving patient outcomes.
